# Modeling Combinations of Pre-erythrocytic *Plasmodium falciparum* Malaria Vaccines

**DOI:** 10.4269/ajtmh.14-0767

**Published:** 2015-12-09

**Authors:** Andrew S. Walker, José Lourenço, Adrian V. S. Hill, Sunetra Gupta

**Affiliations:** Department of Zoology, University of Oxford, Oxford, United Kingdom; The Jenner Institute, University of Oxford, Oxford, United Kingdom

## Abstract

Despite substantial progress in the control of *Plasmodium falciparum* infection due to the widespread deployment of insecticide-treated bed nets and artemisinin combination therapies, malaria remains a prolific killer, with over half a million deaths estimated to have occurred in 2013 alone. Recent evidence of the development of resistance to treatments in both parasites and their mosquito vectors has underscored the need for a vaccine. Here, we use a mathematical model of the within-host dynamics of *P. falciparum* infection, fit to data from controlled human malaria infection clinical trials, to predict the efficacy of co-administering the two most promising subunit vaccines, RTS,S/AS01 and ChAd63-MVA ME-TRAP. We conclude that currently available technologies could be combined to induce very high levels of sterile efficacy, even in immune-naive individuals.

## Introduction

It is estimated that, since 2000, global malaria-associated mortality has declined by 47%, largely due to increased distribution of insecticide-treated bed nets and artemisinin-based combination therapies.^1^ However, it is widely accepted that vaccine research must continue as the international community pushes for elimination,^2^ especially in light of recently emerged artemisinin resistance.^3^ The malaria parasite has a complex lifecycle, each stage of which is the target of current vaccine research, from the initial sporozoite inoculum and subsequent liver stages to erythrocytic infection; vaccines are also being developed against the sexual stages with the aim of blocking transmission.

Pre-erythrocytic (PE) parasite stages have been targeted using either whole parasite approaches or recombinant vaccines.^4^,^5^ Among the latter, the RTS,S subunit vaccine has shown moderate, short-term efficacy, with a large-scale phase III clinical trial completed in 2014.^6^–^8^ Protection is conferred mainly by anti-circumsporozoite protein (CSP) antibodies, although a contribution from CSP-specific CD4^+^ T cells that help antibody production cannot be ruled out.^9^ An efficacy of around 50% (95% confidence interval [CI]: 32.9–67.1%) was shown in a phase IIa controlled human malaria infection (CHMI) trial when combined with AS01B, a liposome-based adjuvant system.^10^ Another PE subunit vaccine ME-TRAP, which induces CD8^+^ T-cell responses against infected hepatocytes, has been shown to provide 21% sterile protective efficacy in a phase IIa CHMI trial when administered by a prime-boost regimen (ChAd-MVA).^11^ Both of these vaccines produce a substantial delay in the time to blood-stage infection among those subjects who do not show sterile protection, and levels of reduction in parasite numbers to achieve such a delay have been estimated to be in excess of 95% for both RTS,S^12^ and ME-TRAP.^11^

It has been proposed that combinations of vaccines acting via distinct biological mechanisms could act synergistically.^13^–^18^ The aforementioned vaccines, each with well in excess of a 90% reduction in parasite numbers^11^,^12^ and discrete methods of action, could prove a potent combination. Such an effect has previously been noted in a murine model, in which a pair of T-cell- and antibody-inducing vaccines, each with around 30–35% sterile efficacy when administered alone, elicited 90% sterile efficacy upon their combination.^16^ Here, we use a mathematical model to investigate the effects of combining antimalarial vaccines acting at different PE stages of the life cycle. We used data from control subjects within phase IIa CHMI trials^10^,^11^ to parameterize the within-host dynamics of the PE stages of *Plasmodium falciparum* and derive measures of the effects of RTS,S and ME-TRAP on infection using data from subjects who have received these vaccines. We show that high levels of sterile protection may be obtained by two vaccines which each show far lower efficacy when administered alone.

## Methods

### The model.

Within-host parasite dynamics are modeled by the following system of equations describing the rates of change in numbers of infected hepatocytes (H) and merozoites (M), with parameters as described in Table 1:

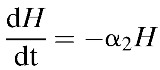


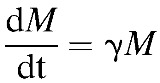


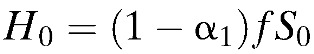


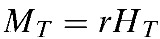


Here, *H*_*0*_ and *M*_*T*_ specify starting conditions for the respective populations, with *M* = 0 when *t* < *T*, where *T* = incubation period within the liver. There are several stochastic events in the development of malaria infection, made apparent in CHMI trials by differences between individuals in the time taken to reach defined thresholds of parasitemia. The model captures this behavior by incorporating inter-individual variation in the sporozoite inoculum (*S*_0_), probability of liver invasion (*f*), incubation period (*T*), and erythrocytic growth rate (γ). We assume that an individual receives *n* bites, where the size of each sporozoite inoculum, *S*_i_ (such that the total initial inoculum *S*_0_ = Σ*S*_i_), is sampled from a negative binomial distribution obtained by fitting a negative binomial model to counts of parasites inoculated per-bite by anopheline mosquitoes.^19^ The proportion of sporozoites invading liver cells (*f*) is drawn from a Poisson distribution with shape parameter *ɛ* = *S*_0_/λ, where λ is a modifier of sporozoite success. Vaccine-induced anti-sporozoite immune responses reduce the probability of invasion by (1 − α_1_), while infected hepatocytes are removed by a liver-stage vaccine at a rate α_2_. Intrahepatocytic development takes approximately 7 days, and at the end of this period (*T*), the merozoite population size is set to *rH*_*T*_, where *r* is the number of merozoites released per hepatocyte. Erythrocytic growth occurs at a rate γ, sampled from a Gaussian distribution with a given mean and standard deviation (SD; γ_mean_ and γ_sd_, respectively).

The model was implemented in MATLAB R2013b (The MathWorks, Inc., Natick, MA), and the ODE system solved using a non-stiff Runge-Kutta solver, ode45.

### Model fitting to trial data.

The two studies used to parameterize the model^10^,^11^ utilized similar challenge protocols, and there were no significant differences in time to patency (*t*_df_ = 1.5409_34_, *P* = 0.1326) between the two trials, nor was there a difference between either study and an additional study investigating time to patency following *Anopheles* bites.^20^ Similarly, there was no significant difference between the times to quantitative real-time polymerase chain reaction (qPCR) detection in the Ewer and Lyke studies (*t*_df_ = 1.320_22.755_, *P* = 0.2002); qPCR data was not available for the RTS,S CHMI study.^10^

The combination of parameters that provides the best fit between the ODE model and the CHMI trial data was determined using a stepwise Markov chain Monte Carlo (MCMC) methodology,^21^ detailed in Supplemental Methods. Each run of the model outputs the time from sporozoite inoculum to qPCR and blood slide detection of merozoites; these values are compared with CHMI data. Threshold levels of parasitemia were computed on the basis that 1) qPCR detection methods can reliably detect 20 parasites/mL of blood^22^ and, given ∼4.7 L of blood in an adult human, this equates to 94,000 parasites in a single host and 2) blood slide methods generally detect parasitemia when there are between 20 and 50 parasites/μL^23^: an estimated mean of 35 was therefore used, equating to 1.645 × 10^8^ parasites across the human host.

First, a baseline model was parameterized (Supplemental Methods) using CHMI data from non-vaccinated individuals. The effects of administering two vaccines in combination were assessed by running the model 500 times under different parameters describing the proportional reduction in successful sporozoite infection and maturation of infected hepatocytes. These particular parameters for RTS,S and ME-TRAP were obtained by fitting the baseline model to CHMI data on vaccinated individuals.

The model was subjected to a sensitivity analysis by fixing (baseline model and vaccine) parameters at the values found by the MCMC procedure (Supplemental Table 2.), and then varying each parameter independently about a Gaussian distribution with arbitrary SD. For each parameter value, the least squares distance of the model output to the trial data was calculated.

## Results

### Baseline within-host dynamics.

**Figure 1.**
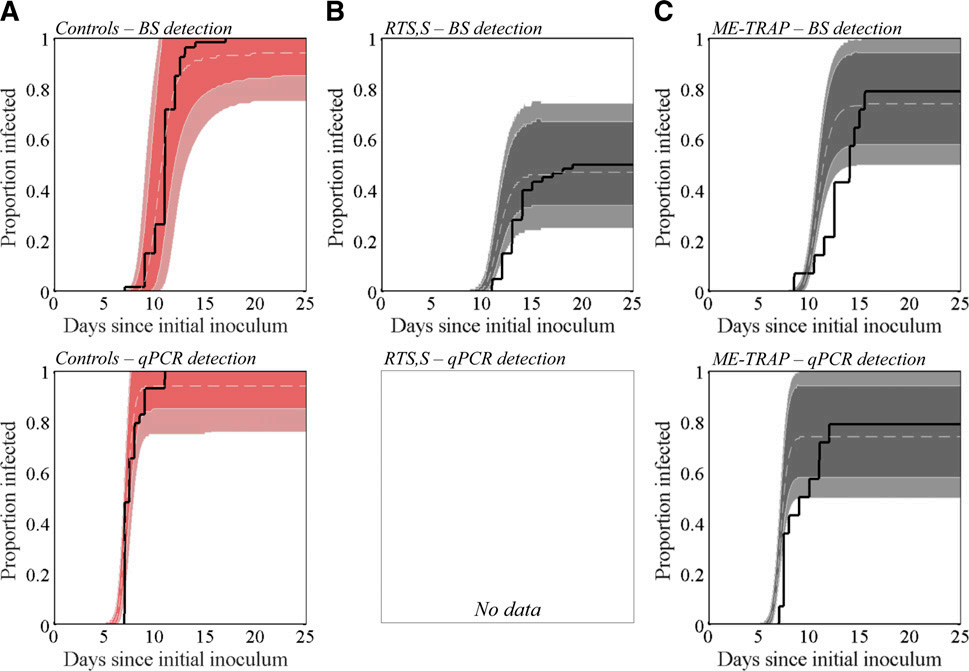
Survival curves showing the proportion of individuals who have reached the thresholds of blood-stage parasitemia required for detection by either blood slide (BS) or quantitative real-time polymerase chain reaction (qPCR) analysis—no qPCR data were available for the RTS,S/AS01B study analyzed. Solid lines represent data from CHMI trials that was used to fit (**A**) the baseline (no vaccine), (**B**) RTS,S/AS01B,^10^ and (**C**) ChAd63-MVA ME-TRAP^11^ models. The dashed white lines represent the median of all accepted chain steps of the Markov chain Monte Carlo (MCMC) protocol (post burn-in time), and dark and light shaded areas represent 90% and 99% credible intervals, respectively.

We obtained a mean time to patency by blood slide analysis of 11.046 days (SD = 1.205) as compared with 11.037 (SD = 1.512, *N* = 53) among the controls in the CHMI trials used in this study (Figures 1A

and 2A

**Figure 2.**
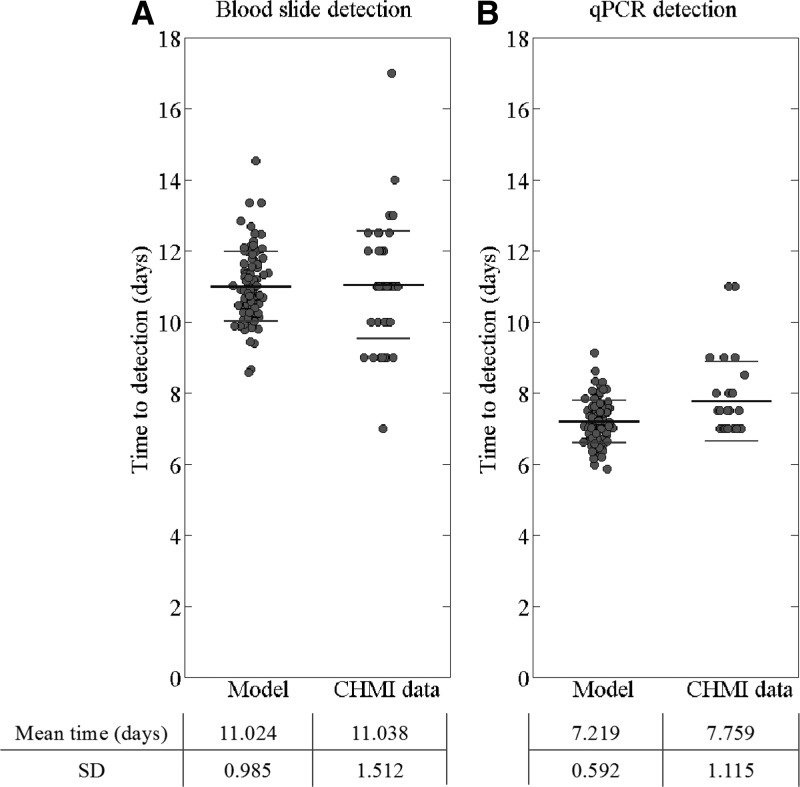
Time to (**A**) blood slide patency and (**B**) detection by quantitative real-time polymerase chain reaction (qPCR) in CHMI trials^10^,^11^,^20^ and the model for control (non-vaccine recipient) individuals. Model data is based on 500 runs with parameters fixed at the values found by Markov chain Monte Carlo (MCMC) fitting, 100 randomly selected runs were plotted. Mean and ± 1 standard deviation (SD) are shown (black/gray lines, respectively).

). The mean time to the qPCR detection threshold of parasitemia in the model was 7.219 days (SD = 0.592), compared with 7.759 days (SD = 1.115, *N* = 29) in the CHMI trials (Figures 1A and 2B and Supplemental Table 1).

For completeness, we performed an additional sensitivity analysis (Supplemental Figure 1A–D) by resampling each parameter independently from a normal distribution with a mean of the value found by fitting. We found the goodness of fit of the model to be dependent on all parameters and, as expected, to maximize around the values found by the MCMC.

### Within-host dynamics in single vaccine recipients.

We obtained a sterile protective efficacy of 53.35% (SD = 5.20%) for RTS,S/AS01 compared with 50% (95% CI = 32.9–67.1%) reported by Kester and others^10^ (Figure 1B). In those individuals who were not protected, the mean time to patency by blood slide analysis in the model was 11.898 days (SD = 1.222), compared with the CHMI result of 13.567 days (95% CI = 9.78–17.37; Supplemental Figure 2A and Supplemental Table 1). Time to qPCR detection in the model was 7.896 days (SD = 0.603); this cannot be compared with trial output, as qPCR data were not available in this trial (Figure 1B and Supplemental Figure 2B). This corresponds to a proportionate reduction in successful sporozoite numbers (α_1_) of 0.901 (Supplemental Table 2). Overall, the fit of the model to the RTS,S (α_1_) data is better than to ME-TRAP (α_2_) data (Figure 4), which may in part be due to only blood slide patency data available for RTS,S, whereas both blood slide and qPCR data were available for ME-TRAP.

ChAd-63-MVA ME-TRAP elicited 22.72% efficacy (SD = 4.26%) in the model as compared with 21.4% (95% CI = 3.2–46.0%) in the study by Ewer and others.^11^ The mean time to blood slide and qPCR detection in non-protected individuals in the model were 11.008 days (SD = 1.054) and 7.208 days (SD = 0.76), respectively, compared with 12.82 days (95% CI = 8.72–6.91) and 8.91 days (95% CI = 5.41–12.41) in the vaccine trial (Figure 1C, Supplemental Figure 2C, D, and Supplemental Table 1). This corresponds to a proportionate reduction in infected hepatocytes (1 − exp(−α_2_ × T)) of 0.780 (Supplemental Table 2).

### Effects of combining vaccines.

**Figure 3.**
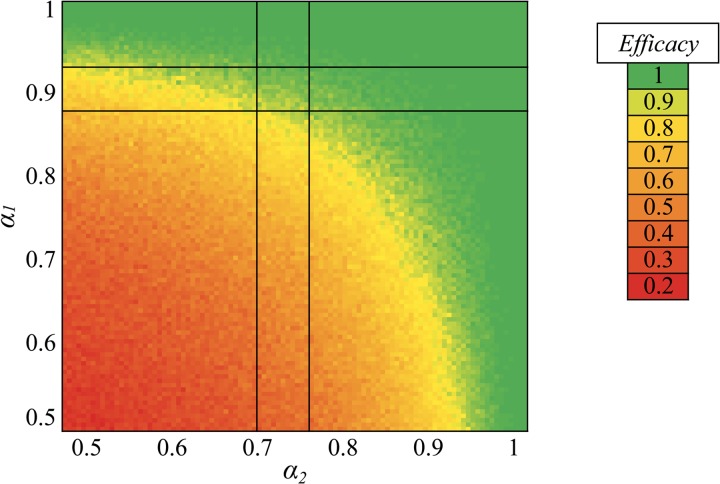
Proportional sterile protective efficacies of combinations of vaccines, with color indicating proportional sterile efficacy from green (total protection) to red (zero protection). The sets of intersecting lines indicate the calculated potencies of RTS,S/AS01B (horizontal line^10^) and ChAd63-MVA ME-TRAP (vertical line^11^) vaccines when used in monotherapy in CHMI trials: modeling their combination gives an efficacy between 93% and 99%.

Sterile protective efficacies of combinations of vaccines acting at sporozoite and liver stages were evaluated by varying α_1_ and α_2_ to reflect a proportionate reduction in parasite numbers ranging from 0.5 to 1 (Figure 3

**Figure 4.**
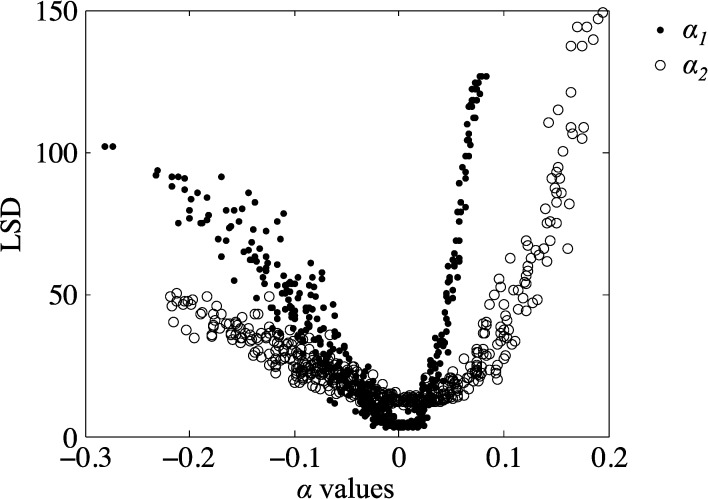
The effect of independently varying α_1_ (•) and α_2_ (○) on the least squares distance (LSD) between the model and the data, with all other parameters fixed. Each point (*N* = 500) represents the mean of 100 runs of the ODE model.

). We found that the parameters identified for RTS,S and ME-TRAP from the CHMI data conferred very high levels of sterile protection when the vaccines were combined (Figure 3, intersection of double lines): 97.51% (SD = 1.52%) of infections were prevented in 500 runs of the model with α values fixed at the values found by MCMC fitting. A small increase in percentage killed for either vaccine would effectively yield sterile protection in combination with the other, but only a very modest rate if used on its own.

## Discussion

An effective vaccine against *P. falciparum* malaria is widely accepted as an essential step toward eradication of the disease. In this study, we explore the possibilities for synergistic efficacy in combinations of PE vaccines. Underpinning this is the requirement of vaccine-induced immune responses to elicit sterile protection, since even in individuals in whom all but a few parasites are eradicated, infection is likely to occur, albeit with a slight delay. Previous studies have estimated two PE subunit vaccines currently in development, ChAd63-MVA ME-TRAP and RTS,S/AS01, to independently eradicate in excess of 90% of parasites,^11^,^12^^,^ and yet sterile efficacies remain moderate to modest.^8^,^10^,^11^,^24^,^25^ We suggest that combinations of vaccines with such strong parasite-killing effects will be significantly more efficacious than the aforementioned, although highly promising, single-vaccine approaches.

Our estimates of the combined efficacy of ChAd63-MVA ME-TRAP and RTS,S/AS01 would be sufficient, at high coverage, to eliminate malaria in areas where the transmission potential (*R*_0_) of malaria is moderate^26^ (∼5), and would extend to areas of much higher transmission by even very slightly increasing the rate of parasite killing for either vaccine. There is considerable debate concerning the measurement of *R*_0_ for malaria,^27^,^28^ but methods that account for antigenic diversity and low rates of development of natural immunity^28^ suggest maximum values that fall well within the range of possible elimination associated with the combined efficacy of ChAd63-MVA ME-TRAP and RTS,S/AS01. The duration of efficacy remains a problem in achieving the goal of elimination: to date, 13 PE and nine erythrocytic vaccines have entered clinical trials,^5^ but very few have been found to demonstrate lasting protective efficacy in humans, despite high antibody titers or T-cell levels.^4^ Thus, although our model supports the notion that a highly efficacious PE subunit vaccine may well be within reach, control of malaria is likely to be sustained only when used in conjunction with other tools^5^ such as bednets^29^ and fungal biopesticides.^30^

A paradox arises when trying to fit the model to data on infection dynamics among recipients of ME-TRAP (Figure 1C) in that the recorded delay to thresholds of parasitemia require less than one liver cell's worth of merozoites to be released. This suggests that ME-TRAP, as well as inducing total destruction of liver cells, may cause partial disruption of others, allowing them to release a partial load of merozoites. Partial release of merozoite load may also be a feature of natural infection, as noted by Bejon and others in trying to replicate infection dynamics among CHMI controls.^12^ There are several potential mechanistic explanations for this phenomenon. It appears that hepatocytes do not “burst” per se, rather, vesicles called merosomes, each containing 100–200 merozoites, bud from the infected cell,^31^ and it has been shown that merosome exit from the liver can be affected by inflammatory immune responses restricting blood flow.^32^ Furthermore, the inherent stochasticity in the erythrocytic invasion process^12^ may be partially responsible for the range in times to qPCR detection and patency by blood slide analysis seen in CHMI studies.^33^ Finally, it is likely that there is heterogeneity in the spread of parasites across the body. For example, release of the merosome cargo has been shown in a murine model to occur mostly in the lungs^34^: parasites are released into deep vasculature, whereas blood for qPCR detection and thick smear analysis is taken from more peripheral sources.

We have assumed that there is no interaction between the T-cell- and antibody-mediated responses to ChAd63-MVA ME-TRAP and RTS,S/AS01. An important consideration in future work will be, when combining vaccines, whether interactivity exists between the mechanisms by which they act. Furthermore, certain candidate vaccines may affect more than one stage in the life cycle: for example, the blood-stage candidate apical membrane antigen 1 (AMA-1), also plays a role in hepatocyte invasion^35^ and has demonstrated promising efficacy correlated with cell-mediated immunity when combined with a PE antigen.^36^ Our model provides a platform to investigate the non-additive effects of the various combinations of vaccines that are likely to be tested in the near future in our ongoing battle against malaria.

## Supplementary Material

Supplemental Datas.

## Figures and Tables

**Table 1:** Parameters and initial conditions in the model

Parameter	Explanation	Value
α_1_	Vaccine-induced modifier of sporozoite invasion probability: α_1,min_ = 0, α_1,max_ = 1	MCMC fitted
α_2_	Vaccine-induced rate of removal of infected hepatocytes: α_2,min_ = 0, α_2,max_ = 1	MCMC fitted
μ	Rate of sporozoite loss, set such that sporozoites are removed from system at a realistic rate	20
*T*	Liver incubation time: time from sporozoite inoculum to merozoite release	Selected from Gaussian distribution, mean = 7 days, SD = 0.5 days
*r*	Successful merozoites per hepatocyte	10,000
*f*	Proportion of sporozoites that successfully invade hepatocytes: if, *f* > 1, *f* = 1	Stochastically selected from Poisson distribution (see Methods)
λ	Shape determinant for Poisson cumulative density function from which sporozoite success rate is stochastically sampled: λ_max_ = 10; λ_min_ = 1	MCMC fitted
*S*_0_	Initial inoculum size, based on sum of five samples (five bites) from negative binomial distribution	–
*P*	Success parameter for sampling from negative binomial to give per bite inoculum size	MCMC fitted
*R*	Shape parameter of inoculum size negative binomial^19^	0.246
γ_mean_	Mean of EGR: γ_mean,max_ = 5; γ_mean,max_ = 0	MCMC fitted
γ_sd_	SD of EGR: γ_sd,max_ = 1.25; γ_sd,max_ = 0	MCMC fitted
γ	EGR: if γ < 0 then γ = 0	Sampled from Gaussian of mean γ_mean_ and SD γ_sd_

EGR = erythrocytic growth rate; MCMC = Markov chain Monte Carlo; SD = standard deviation.
