# Supplementary material for: Modeling Combinations of Pre-erythrocytic Plasmodium falciparum Malaria Vaccines
Source: Am J Trop Med Hyg. 2015 Dec 9;93(6):1254–9. doi: 10.4269/ajtmh.14-0767 (PMC4674243; doi:10.4269/ajtmh.14-0767)
Supplement: Supplementary file 1 [file SD5.pdf]

SUPPLEMENTAL METHODS

Each step of the Markov chain Monte Carlo (MCMC) routine effectively represents one clinical trial, with 100 runs of the ODE model completed with parameters at values set to the current MCMC state: the proportions of these runs that reach the two parasite density thresholds (time to blood slide/quantitative real-time polymerase chain reaction [qPCR]) comprise the main output of the model. The least squares distance (LSD) between this output ($\chi^2(O(Y))$ and the CHMI trial data ($\chi^2(O(M^t))$ was calculated, and the likelihood that the given parameter set describes the observed trial data was computed using the expression:

$$\rho = e^{\chi^2(O(Y)) - \chi^2(O(M^t))}$$

where ρ is the MCMC probability of acceptance, O the ODE model output from either Y, the proposed parameter set, or M^t, the accepted parameter set at time t. Increases in LSD are assumed to exponentially decrease the probability of acceptance. A value between 0 and 1 is selected from a uniform distribution, and if this value is less than ρ, the proposed parameter set is accepted, thus making $M^{t+1} = Y$.

To establish a baseline parameter set, the ODE model was fit to data[10,11,20] for individuals who did not receive a vaccine (parameters marked "MCMC fitted" in Table 1). First, to establish a primary parameter set, a set of independent MCMC chains were run with random starting conditions. After convergence, the parameter distributions from each chain were used to inform an initial state for a single MCMC chain of 500,000 steps, with all accepted parameter sets gathered for further analysis. From this single chain an optimum parameter set was found by calculating the median values of the 100,000 best fitting chains (by lowest LSD).

The previously sought parameters were then fixed, and the model rerun for two independent sets of 500,000 MCMC steps, now fit to data from vaccine recipients in each of the two CHMI trials: α_1 was fit to data from the RTS,S recipients and α_2 subsequently fit for ME-TRAP recipients. As only a single parameter (α_1 or α_2) was varied at a time, these chains converged more quickly than the baseline MCMC model fitting (four parameters).

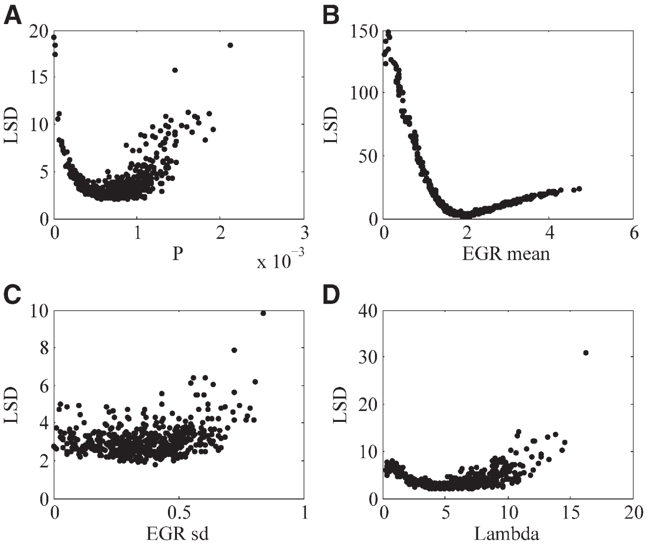

SUPPLEMENTAL FIGURE 1. Results of a sensitivity analysis conducted on the model, demonstrating change in least squares distance (LSD) between the model and the controlled human malaria infection (CHMI) trial data as a result of varying single parameters about a mean set by the Markov chain Monte Carlo (MCMC) procedure. The probability parameter of the negatively binomial inoculum distribution (**A**), γ_{mean} (**B**), γ_{sd} (**C**), and λ factor (**D**) are varied in a system with no vaccine.

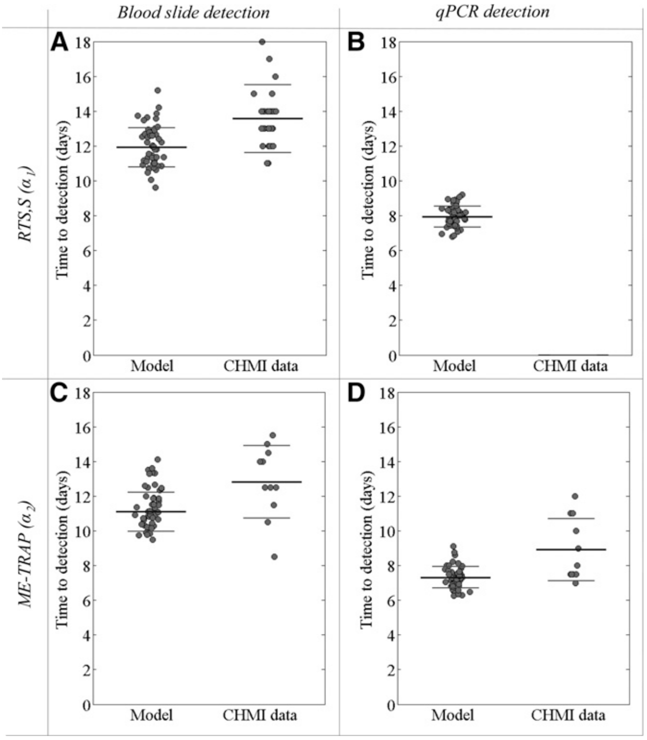

SUPPLEMENTAL FIGURE 2. Distributions of time to (**A** and **B**) blood slide patency and (**B** and **D**) quantitative real-time polymerase chain reaction (qPCR) thresholds of blood-stage parasitemia under vaccination with (**A** and **B**) RTS,S and (**C** and **D**) ME-TRAP, comparing the controlled human malaria infection (CHMI) trial data and the model outputs. Model data show 50 points randomly selected from 500 runs of the model. Horizontal lines indicate means and standard deviations of all outputs/CHMI trial groups. No qPCR data were available for the RTS,S CHMI trial—the model was solely fit to blood slide patency data.

Supplemental Table 1

Times to blood slide detection and qPCR detection thresholds under treatment by RTS,S or ME-TRAP in both the model and CHMI data.

		Blood slide detection		qPCR detection	
		Model	CHMI	Model	CHMI
RTS,S	Mean	11.921	13.567	7.949	n/a
	SD	1.276	1.942	0.605	n/a
ME-TRAP	Mean	11.100	12.818	7.317	8.909
	SD	1.266	2.089	0.627	1.786

Supplemental Table 2

Parameter values estimated by the MCMC fitting procedure

			Quantile		
		Parameter	0.05	0.5	0.95
Baseline model	P	Inoculum negative binomial parameter	0.000567	0.00111	0.00555
	λ	Sporozoite success modifier	1.246	5.763	9.003
	γ_{mean}	Mean of EGR	1.569	2.175	3.194
	γ_{sd}	SD of EGR	0.0652	0.546	1.143
Under vaccination	α_1	Vaccine-induced modifier of sporozoite invasion probability	0.854	0.907	0.935
	α_2	Vaccine-induced rate of removal of infected hepatocytes	0.116	0.216	0.305

EGR = erythrocytic growth rate; MCMC = Markov chain Monte Carlo; SD = standard deviation.
